# A Cohort Study of Exposure to Antihyperglycemic Therapy and Survival in Patients with Lung Cancer

**DOI:** 10.3390/ijerph17051747

**Published:** 2020-03-07

**Authors:** Edvardas Danila, Donata Linkevičiūtė-Ulinskienė, Rolandas Zablockis, Vygantas Gruslys, Saulius Cicėnas, Giedrė Smailytė

**Affiliations:** 1 Clinic of Chest Diseases, Immunology and Allergology of Faculty of Medicine of Vilnius University, Santariskiu g. 2, Vilnius LT-08661, Lithuania; rolandas.zablockis@santa.lt (R.Z.); vygantas.gruslys@santa.lt (V.G.); 2 Center of Pulmonology and Allergology of Vilnius University Hospital Santaros Klinikos, Santariškių g. 2, Vilnius LT-08661, Lithuania; 3 Department of Pathology, Forensic Medicine and Pharmacology, Institute of Biomedical Sciences, Faculty of Medicine, Vilnius University, Čiurlionio g. 21, Vilnius LT-08661, Lithuania; linkeviciutei@gmail.com; 4 Department of Thoracic Surgery and Oncology, National Cancer Institute, Santariškių g. 1, Vilnius LT-08660, Lithuania; saulius.cicenas@nvi.lt; 5 Clinic of Internal Diseases, Family Medicine and Oncology, Faculty of Medicine, Vilnius University, Santariškių g. 2, Vilnius LT-08661, Lithuania; 6 Laboratory of Cancer Epidemiology, National Cancer Institute, P. Baublio g. 3b, Vilnius LT-08406, Lithuania; giedre.smailyte@nvi.lt; 7 Department of Public Health, Institute of Health Sciences, Faculty of Medicine, Vilnius University, Čiurlionio g. 21, Vilnius LT-08661, Lithuania

**Keywords:** diabetes, insulin, lung cancer, metformin

## Abstract

We evaluated the effect of antihyperglycemic therapy on the survival of patients with lung cancer (LC). The analysis included patients with LC and concomitant type 2 diabetes. 15,929 patients were classified into five groups: metformin users, insulin users, metformin and insulin users, sulphonylurea users and non-diabetic group. A multivariate analysis showed that exposure to either metformin or to insulin was associated with a lower risk of LC-specific mortality, and this approached statistical significance (HR 0.82, 95% CI 0.72–92 for metformin and HR 0.65, 95% CI 0.44–95 for insulin). When deaths from all causes were considered, only metformin exposure was associated with a significantly lower risk of death (HR 0.82, 95% CI 0.73–0.92). Users of sulphonylurea were at a higher risk of LC-specific and overall mortality (HRs 1.19, 95% CI 0.99–1.43 and 1.22, 95% CI 1.03–1.45). Our study shows a positive effect of metformin on the survival of patients with LC. Moreover, our results show that exposure to insulin was associated with a lower risk of LC-specific mortality, but not with deaths from all causes. The study results suggested that users of sulphonylurea may be at a higher risk of LC-specific and overall mortality.

## 1. Introduction

Lung cancer, in both sexes combined, was the most commonly diagnosed cancer (2.1 million new cases, 11.6% of the total cancer cases) and the leading cause of cancer death (1.8 million deaths, 18.4% of the total cancer deaths) in 2018 [1]. Although lung cancer occurs in adult patients of all ages, it predominates among people over 50 years old, most often at the age of 60–75 years [2]. Unsurprisingly, comorbidities are common in patients with lung cancer, including diabetes. Approximately 6–18% of lung cancer patients also have diabetes [3,4].

Diabetes itself has a negative effect on lung cancer prognosis by increasing the risk of cardiovascular events, complications after surgery and infection [3,5]. A worse lung cancer prognosis in diabetes patients may be caused by different biological mechanisms related to hyperglycemia, hyperinsulinemia and inflammation, which have been implicated in carcinogenesis progression and result in tumor cell proliferation and metastases [6,7,8]. Though an association between antihyperglycemic therapy, lung cancer and its prognosis was reported [9,10], the results are conflicting [11,12,13,14]. 

Metformin and insulin are standard drugs for the treatment of diabetes [15]. Some studies [16,17] showed that metformin could improve the outcome in lung cancer, but others [18,19] did not find a protective effect of metformin. Though insulin treatment also showed a survival advantage [20], there is still little published data on the insulin effect on the survival of patients with lung cancer. 

Considering that comorbidity is increasing due to the aging population [3] and that lung cancer is becoming a chronic disease [21], it is important to know the long-term effects of concomitant medication on lung cancer outcomes.

The main objective of our study was to evaluate the effect of metformin-based therapy and insulin-based therapy on the survival of patients with lung cancer.

## 2. Methods

### 2.1. Data Sources & Study Design

The data for this study came from the Lithuanian Cancer Registry and the National Health Insurance Fund (NHIF) database. Individual records of cancer patients from the Cancer Registry were joined with data, available from the NHIF, on other diagnoses, such as type 2 diabetes (ICD-10-AM code E11), healthcare services and prescriptions of reimbursed drugs. 

We identified all patients newly diagnosed with primary invasive lung cancer (International Classification of Diseases Australian modification, ICD-10-AM codes C33 and C34) between January 1, 2001 and December 31, 2013 from the Cancer Registry. 

**Figure 1:**
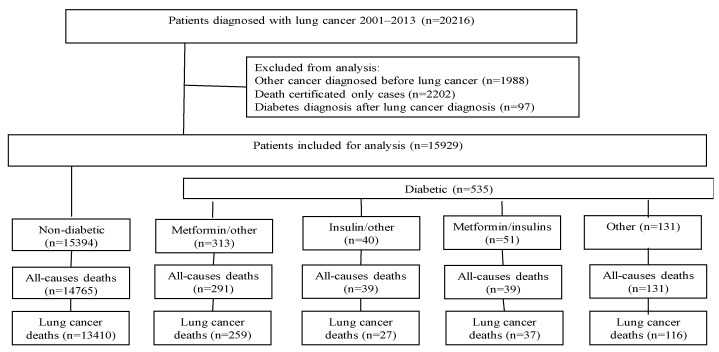
Study flow chart of lung cancer patients.

Patients identified as death certificated-only cases, those with a malignancy prior to lung cancer and those with diabetes diagnosed after the lung cancer diagnosis were excluded from the cohort. A flow chart which shows the selection process of the cohort is presented in Figure 1.

### 2.2. Exposure Definition

The members of the cohort were classified into five groups. Four groups included type 2 diabetes patients (referred to as diabetes in this study) according to treatment: metformin and other medication users; insulin and other medication (except from metformin) users; metformin and insulin users; sulphonylurea users; and a non-diabetic group. 

The diabetes groups only included patients who were on antihyperglycemic medications. This restriction was applied to make sure that all the patients had diabetes. Patients without a diabetes diagnosis in the NHIF database were classified as non-diabetic. Exposure to antihyperglycemic medications was identified from linked prescription data. Exposure (yes/no) was defined according to whether or not the individual had a supply of antihyperglycemic medications available at any point. 

For metformin users, the cumulative dose (mg) of metformin was calculated from the NHIF database to assess a dose–response relationship. This was stratified into three exposure categories by tertiles.

### 2.3. Outcomes

The primary outcome was lung cancer-specific survival. A secondary analysis evaluated the overall survival. The cohort entry date was the date of the lung cancer diagnosis. Patients were followed up with reference to the vital status until 31 December 2017.

The survival outcomes in patients with lung cancer were compared between non-diabetic and diabetic patients by antihyperglycemic medication user groups. Lung cancer-specific survival was the primary outcome, measured from the date of lung cancer diagnosis to the date of death due to lung cancer or the end of the follow-up. Patients who were not deceased or who died of causes other than lung cancer were censored at the end of the follow-up or the date of death, respectively. The overall survival was analysed as a secondary outcome and defined as the period from the date of diagnosis of lung cancer to the date of death or the end of the follow-up. For this secondary outcome, only those patients who were not deceased were censored at the end of the follow-up.

### 2.4. Covariates

Patients were categorized by sex, age at diagnosis (<59, 60–69, 70–79 and 80+ years), tumour histology and stage at diagnosis (TNM – Tumour, Node, Metastasis Classification of Malignant Tumours). The histology of tumours was grouped according to ICD-O-3 codes into the following five categories: small cell lung carcinoma (ICD-O-3 codes 8041–8045), squamous cell lung carcinoma (8050–8082), adenocarcinoma (8140–8191, 8201–8221, 8250–8300, 8312–8420, 8440–8550), other and unspecified. 

### 2.5. Statistical Analysis

The patient demographic and clinical characteristics were tabulated for all the exposure groups. Kaplan-Meier survival analyses were used for both lung cancer-specific and overall survival. The survival curves were stratified by exposure group and compared using the log-rank test.

Univariate and adjusted Cox proportional hazard models were used to estimate associations between antihyperglycemic medication exposure, and lung cancer-specific survival and overall survival. Hazard ratios (HR) with 95% confidence intervals (CI) were calculated.

Univariate Cox proportional hazard models were used to estimate the hazard ratios and their 95% confidence intervals to compare lung cancer-specific survival differences by sex, age at diagnosis, stage at diagnosis, histology of tumour and diabetes (yes/no). Multivariate Cox proportional hazard models for lung cancer-specific and overall survival included prognostic factors which had a significant impact on survival as determined by a univariate hazard ratio (HR) with a *p*-value < 0.2. 

A statistical analysis was performed with Stata software (version 11.0; StataCorp, College Station, TX, USA).

## 3. Results

### 3.1. Characteristics of the Study Cohort

The patient characteristics for the non-diabetic group and diabetic group are summarised in Table 1. 

During the study period, 15,929 patients diagnosed with incident lung cancer in Lithuania were included in the study. The study group included 83.7% male and 16.3% female lung cancer patients. At presentation, 65.1% of patients were 60–79 years old, 29.6% had adenocarcinoma and 67.8% had tumours diagnosed at stages III and IV. Linkage with the NHIF database yielded 535 patients (3.4%) who were diagnosed with diabetes before lung cancer diagnosis. During the follow-up period, 15,273 (95.9%) lung cancer patients died, and 13,849 patients (86.9%) died of lung cancer. 

### 3.2. Survival Analyses: Lung Cancer-Specific and Overall Mortality

**Figure 2:**
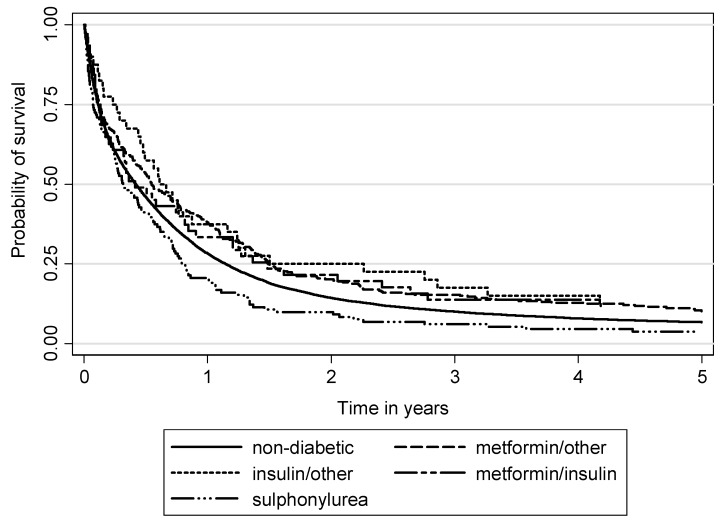
Lung cancer-specific survival between non-diabetic and diabetic patients by antihyperglycemic medication user groups (*p* = 0.0001).

**Figure 3:**
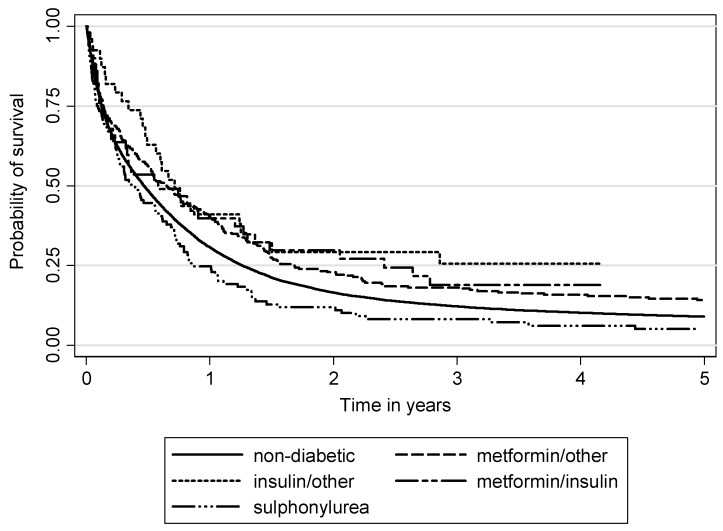
Overall survival between non-diabetic and diabetic patients by antihyperglycemic medication user groups (*p* = 0.0004).

The Kaplan-Meier survival analysis showed differences for lung cancer-specific survival between non-diabetic and diabetic patients by antihyperglycemic medication user groups, with better survival in the groups of insulin and metformin users (Figure 2 and Figure 3). The lowest survival (and lower than in non-diabetic patients) was observed in diabetic patients who were sulphonylurea users.

In the multivariate analysis, after adjustment for sex, age at diagnosis, stage at diagnosis and tumour histology, some of the observed differences became insignificant for both lung cancer-specific and overall mortality (Table 2 and Table 3). Exposure either to metformin or to insulin was associated with a lower risk of lung cancer-specific mortality, and this approached statistical significance (HR 0.82, 95% CI 0.72–92 for metformin and HR 0.65, 95% CI 0.44–95 for insulin, Table 2). When deaths from all causes were considered, only metformin exposure was associated with a significantly lower risk of death (HR 0.82, 95% CI 0.73–0.92, Table 2). Users of sulphonylurea were at a higher risk of lung cancer-specific and overall mortality (HRs 1.19, 95% CI 0.99–1.43 and 1.22, 95% CI 1.03–1.45). 

We found no differences between different cumulative metformin dose groups in diabetic metformin users in the multivariate analysis after adjustment for sex, age at diagnosis, stage at diagnosis and tumour histology for both lung cancer-specific and overall mortality (Table 4).

## 4. Discussion

In our study, the prevalence of diabetes mellitus (3.4%) in lung cancer patients was lower than in other (8%−16%) studies [3,18,22,23], but closer to the prevalence of diabetes globally [24,25]. Additionally, our study population was comprised of a larger proportion of men. As a result, men were the larger group of the diabetes patient cohort. This is not a surprising result, as lung cancer incidence rates in Lithuania in men are high compared to women (age-standardised rate 53.5 per 100,000 and 7.3 per 100,000, respectively) [26]. Metformin is an oral antidiabetic drug considered as the first choice for the oral treatment of type 2 diabetes [27]. Metformin acts by inhibiting hepatic gluconeogenesis [28], reducing insulin resistance [29] and decreasing inflammatory response [30], thus controlling circulating glucose and presenting the potential antitumor effect [31].

We found that the metformin use was associated with a lower risk of lung cancer-specific mortality (HR 0.82, 95% CI 0.73−0.92, *p* < 0.001) and with a lower risk of death from all causes (HR 0.83, 95% CI 0.7−0.92, *p* = 0.001). Several studies reported similar findings to our study [16,20,32,33,34,35,36,37,38,39,40]. 

Furthermore, in a preclinical study, metformin sensitized lung cancer cells to ionizing radiation, which led to an increased response to radiotherapy [41]. Metformin has also been found to exhibit synergistic effects with chemotherapy and target therapy and has thus been studied as an adjuvant treatment for lung cancer [37,42,43,44,45,46]. 

One important consideration is whether the clinical doses of metformin used to treat diabetes mellitus will reflect preventive effects and an increased survival of patients with lung cancer. We found differences between different cumulative metformin dose groups in diabetic metformin users, with a better survival in the highest tertile of the cumulative dose. However, a multivariate analysis after adjustment for sex, age, disease stage at diagnosis and tumour histology observed that differences became insignificant for both lung cancer-specific and overall mortality. 

Few studies have used dose-response variables to model metformin treatment. Landman et al. have reported that metformin intake was associated with a decrease of cancer mortality and that the effect was dose-dependent. The hazard for cancer mortality decreased by 42% for every 1-g increase in the metformin dose in this study [47]. However, Medairos et al. found no evidence of a significant association between progression-free survival and metformin use with increasing daily dosages [48]. Most metformin drug concentrations reported in laboratory studies are as much as 100-fold higher than metformin concentrations that are clinically effective in the treatment of diabetic patients. Clinical use of metformin at high dose levels could therefore be challenging [49,50]. Further investigations are required to assess the minimal effective concentration, minimal toxic concentration and the effective therapeutic range of metformin in patients with lung cancer. 

Insulin treatment in our study was significantly associated with a lower risk of lung cancer-specific mortality and showed a tendency towards decreased overall mortality. The tendency towards a lower risk of cancer-specific and overall mortality was also observed in the group of both insulin and metformin users, but lacked statistical significance (Table 2 and Table 3).

Data regarding the effects of antihyperglycemic therapy, especially treatment with insulin, on lung cancer outcomes are sparse and conflicting. A prospective study of 2484 female patients by Luo et al. showed that women with lung cancer and diabetes had a significantly increased risk of overall mortality (HR 1.27, 95% CI: 1.07–1.50) and those receiving insulin or metformin also had an increased risk of overall mortality (HR 1.54, 95% CI 1.06–2.23 and HR 1.48, 95% CI 1.09–2.00 respectively) [18]. A cohort study by Ioacara et al. found that the insulin glargine cumulative dose was associated with a lower cancer mortality risk in general (subhazard ratio, SHR 0.94; 95% CI 0.89–0.99, *p* = 0.033), but that lung cancer mortality was not significantly influenced [51]. Two other observational studies by Menamin et al. and Tseng reported no significant association with insulin use and lung cancer-specific mortality [19,52]. 

Insulin is a major regulator of cell metabolism and also a growth factor [53]. Long-acting insulin analogs have been demonstrated to stimulate cancer cell proliferation *in vitro* more than native insulin [54]. However, it is questionable if *in vitro* studies are relevant in humans. Some epidemiologic studies have highlighted concerns for an increased risk of cancer among insulin glarine user [55]. However, methodologic limitations have been pointed out in these analyses, and further investigations have shown conflicting results [56,57,58]. 

The research on the insulin effect on cancer prognosis is also complex. Although it is observed that insulin receptors are often overexpressed in malignant cells, the knowledge of their role in cancer progression and their potential use as prognostic factors is limited [59]. Overexpression of IR (insulin receptor) predicts poor survival in patients with non-small cell lung cancer [60] and breast cancer [61,62]. In vitro, insulin is a growth stimulator that is more potent than IGF-1 in some cancer cells. However, the malignant cell biological response to insulin cannot be predicted only on the basis of the insulin receptor content, as a malignant cell is complex and other factors are activated to promote cancer growth [63,64]. 

In our study, users of sulphonylurea were at a higher risk of lung cancer-specific and overall mortality. Only one study reported the association between sulphonylurea exposure and lung cancer-specific survival. No association was found between sulphonylurea exposure and lung cancer-specific mortality (HR 1.04; 95% CI 0.65–1.66) in this cohort study [19].

This study has got some limitations. First, it is a retrospective study. Therefore, some diabetes mellitus-related factors, such as the duration and complications of the disease, were not collected. However, we clearly discriminated groups according to antihyperglycemic medication users. Moreover, metformin cumulative doses were known and used in the study data statistical analysis. Second, the smoking status and other concomitant diseases of patients were not clarified. It was not possible to collect this information. Third, the types of lung cancer treatment were not assessed due to the study format, as it was not possible to clarify this information. However, lung cancer treatment in Lithuania adheres to international standards, with the exception of immunotherapy, which was not widely available in the study period. 

## 5. Conclusions

The results of our study confirmed the findings of some other reports about the positive effect of metformin on the survival of patients with lung cancer. Metformin may lower the risk of deaths from all causes and lung cancer-related death in lung cancer patients with type 2 diabetes. However, some questions, such as metformin dose-related and exposure time effects, still need to be resolved in future studies. 

Our results show that exposure to insulin was associated with a lower risk of lung cancer-specific mortality, but not with deaths from all causes. Moreover, the study results suggested that users of sulphonylurea may be at higher risk of lung cancer-specific and overall mortality. Future investigations are required to verify these findings.

## Figures and Tables

**Table 1:** Demographic and clinical characteristics of patients with lung cancer, by diabetes status and antihyperglycemic medication use.

Characteristics	Non Diabetic		Metformin and Other Medication Users		Insulin and Other Medication Users		Metformin and Insulin Users		Sulphonylurea Users	
	N	%	N	%	N	%	N	%	N	%
Total	15,394	100.0	313	100.0	40	100.0	51	100.0	131	100.0
Sex										
Male	12,943	84.1	222	70.9	34	85.0	35	68.6	95	72.5
Female	2451	15.9	91	29.1	6	15.0	16	31.4	36	27.5
Age at diagnosis										
<60	4010	26.0	53	16.9	6	15.0	3	5.9	13	9.9
60–69	5315	34.5	123	39.3	18	45.0	17	33.3	39	29.8
70–79	4663	30.3	103	32.9	15	37.5	26	51.0	58	44.3
80+	1406	9.2	34	10.9	1	2.5	5	9.8	21	16.0
TNM stage										
I	1136	7.4	22	7.0	4	10.0	3	5.9	9	6.9
II	2004	13.0	37	11.8	8	20.0	8	15.7	23	17.6
III	4876	31.7	91	29.1	11	27.5	13	25.5	37	28.2
IV	5591	36.3	113	36.1	11	27.5	13	25.5	40	30.5
Missing	1787	11.6	50	16.0	6	15.0	14	27.5	22	16.8
Histological group										
Squamous cell Ca	1837	11.9	36	11.5	4	10.0	3	5.9	13	9.9
Adenocarcinoma	4576	29.7	76	24.3	11	27.5	16	31.4	31	23.7
Small cell Ca	2411	15.7	57	18.2	8	20.0	7	13.7	25	19.1
Other	1902	12.4	34	10.9	4	10.0	6	11.8	10	7.6
Unspecified	4668	30.3	110	35.1	13	32.5	19	37.2	52	39.7

**Table 2:** HR and 95% CI of the association of diabetes, antidiabetic medications use and lung cancer-specific mortality.

Variable	Multivariate-Adjusted HR † (95% CI)	*p*-Value
Sex		
Male	1.00	ref.
Female	0.75 (0.71-0.78)	<0.001
Age at diagnosis		
<60	1.00	ref.
60–69	1.20 (0.14–1.25)	<0.001
70–79	1.34 (1.28–1.40)	<0.001
80+	1.35 (1.26–1.45)	<0.001
TNM stage		
I	1.00	ref.
II	1.61 (1.47–1.76)	<0.001
III	2.82 (2.61–3.06)	<0.001
IV	4.68 (4.32–5.07)	<0.001
Missing	2.67 (2.44–2.93)	<0.001
Histological group		
Squamous cell Ca	1.00	ref.
Adenocarcinoma	0.94 (0.88–0.99)	0.024
Small cell Ca	0.88 (0.83–0.94)	<0.001
Other	1.00 (0.93–1.07)	0.923
Unspecified	1.27 (1.19–1.34)	<0.001
Diabetes		
Non diabetic	1.00	ref.
Metformin and other medication users	0.82 (0.72–0.92)	0.001
Insulin and other medication users	0.65 (0.44–0.95)	0.026
Metformin and insulin users	0.84 (0.61–1.16)	0.283
Sulphonylurea users	1.19 (0.99–1.43)	0.060

† Adjusted for all variables shown in the table.

**Table 3:** HR and 95% CI of the association of diabetes, antidiabetic medication use and overall mortality.

Variable	Multivariate-Adjusted HR † (95% CI)	*p*-Value
Sex		
Male	1.00	ref.
Female	0.75 (0.72–0.78)	<0.001
Age at diagnosis		
<60	1.00	ref.
60–69	1.22 (1.17–1.27)	<0.001
70–79	1.39 (1.33–1.45)	<0.001
80+	1.49 (1.39–1.59)	<0.001
TNM stage		
I	1.00	ref.
II	1.52 (1.41–1.65)	<0.001
III	2.52 (2.34–2.70)	<0.001
IV	4.09 (3.80–4.39)	<0.001
Missing	2.42 (2.23–2.63)	<0.001
Histological group		
Squamous cell Ca	1.00	ref.
Adenocarcinoma	0.92 (0.88–0.98)	0.006
Small cell Ca	0.89 (0.83–0.94)	<0.001
Other	0.98 (0.92–1.05)	0.556
Unspecified	1.32 (1.24–1.39)	<0.001
Diabetes		
Non diabetic	1.00	ref.
Metformin and other medication users	0.82 (0.73–0.92)	0.001
Insulin and other medication users	0.85 (0.62–1.16)	0.299
Metformin and insulin users	0.93 (0.70–1.16)	0.614
Sulphonylurea users	1.22 (1.03–1.45)	0.023

† Adjusted for all variables shown in the table.

**Table 4:** HR and 95% CI of the association of metformin use, and lung cancer-specific and overall mortality by cumulative metformin dose.

Cumulative Dose (mg)	Lung Cancer Specific Mortality HR † (95% CI)	*p*-Value	Overall Mortality HR † (95% CI)	*p*-Value
<360,000	1.00	ref.	1.00	ref.
360,000–1,300,000	0.61 (0.44–0.84)	0.003	0.74 (0.54–1.02)	0.07
>1,300,000	1.05 (0.66–1.71)	0.82	1.03 (0.64–1.66)	0.91

† Adjusted for sex, age at diagnosis, stage at diagnosis and tumour histology.
